# Toxicological risk of melamine and cyanuric acid in food and feed

**DOI:** 10.2478/v10102-009-0010-6

**Published:** 2009-06

**Authors:** Pavel Suchý, Eva Straková, Ivan Herzig, Jaroslav Staňa, Renata Kalusová, Markéta Pospíchalová

**Affiliations:** 1 Faculty of Veterinary Hygiene and Ecology, University of Veterinary and Pharmaceutical Sciences, Brno, Czech Republic; 2 Central Institute for Supervising and Testing in Agriculture, Czech Republic

**Keywords:** adulteration of protein feeds, nitrogen compounds, toxicological consequences

## Abstract

From the toxicological point of view, in the last two years melamine and cyanuric acid have become matters of great interest. These substances, especially melamine, have been abused during food and feed adulteration by increasing the content of nitrogen compounds in these products. Melamine and cyanuric acid as individual substances do not pose any serious risk in terms of toxicology. From the point of view of toxicology, it is especially the complex of melamine with cyanuric acid that is important. This complex, also known as the melamine-cyanurate complex, is a cause of human and animal health problems. In this work we present two examples of the incidence of melamine and cyanuric acid in two feed products originating from China. They were rice and the pea concentrates intended for animal nutrition. Protein concentrates can be the main risk factor for food chain contamination with melamine and cyanuric acid, especially those of unknown origin. Feed with a high content of nitrogen compounds and low content of aminoacids can be regarded as particularly suspicious. A comparison of results for determining nitrogen compounds and amines can be used as proof of adulteration of protein feeds. These feeds must be subjected to further analysis to determine melamine and cyanuric acid.

## Introduction

**Melamine (2,4,6-triamino-1,3,5-triazine)** is a heterocyclic compound containing 6 atoms of nitrogen. As a result of its composition, it contains 66% nitrogen in its molecule. It is a raw material in the production of plastics, pesticides, cleaning agents, fertilizers and many other products, e.g. melamine resin. Pure melamine is a very thermostable crystalline substance, rather poorly soluble in aqueous media. That is why it is used in many products as combustion inhibitor. Based on cyromazine, melamine is a metabolite of insecticides. Within the intermedial metabolism in vegetable and animal organisms this pesticide can be converted to melamine. As a pure substance, melamine in small doses is practically non-toxic. The main toxic effect of melamine in the diet is the formation of urinary stones, inflammatory reactions and hyperplasia in the urinary bladder (Ogasawara *et al*., 1995). Melamine crystalluria has been reported in dogs (Bingham *et al*., 2001). There is a different interspecific sensitivity for melamine. Whereas cats and dogs are especially highly sensitive, rats and rabbits are only slightly sensitive: LD_50_ > 3000mg/kg (Lide, 2005). For the total content of melamine and its analogs, the tolerated daily intake is reported to be 0.5mg/kg of body weight. Melamine is absorbed through the digestive tract and when inhaled it is irritating to skin and eye mucous membranes. Acute toxicity leads to acute renal failure. Chronic toxicity is manifested by the formation of urinary stones in kidneys or bladders. There is a suspicion that there are negative effects of melamine on the reproduction system and carcinogenic effects, esp. at high doses.

**Cyanuric acid – 1,3,5-triazine-2,4,6-triol (CNOH)_3_ –** is a cyclic trimer of hydrocyanic acid (HOCN). It is a white crystalline substance produced by thermal decomposition of urea under defined conditions. It can also be obtained by hydrolysis of melamine. It is a component in various disinfectants, herbicides and bleaching agents. Cyanuric acid as a pure substance can be (like melamine) classified as a non-toxic substance. The LD_50_ value for rats is reported as 7 700mg/kg of live weight (OECD, 1999). It causes damage to kidney tissue in the form of necrosis and hyperplasia, and dilatation of kidney tubules, neutrophile infiltration, mineralization and fibrosis also occur. In humans, more than 98% of orally administered cyanuric acid is excreted in the urine within 24 hours (Allen *et al*., 1982).

Particularly hazardous, however, is especially the combination of melamine with cyanuric acid. This complex is formed by melamine cyanurate, which goes on to form insoluble and very dangerous crystals in the microtubular system of kidneys. These crystals can damage tubules; indeed they act as a pathological agent of renal failure. The melamine-cyanurate complex is much more toxic than either melamine or cyanuric acid alone.

## Methods for determination of melamine and cyanuric acid

Modern analytical methods are used to determine melamine and cyanuric acid (Vail *et al*., 2007, Yamamoto *et al*., 2008). The melamine content in analysed samples of vegetable proteins was determined using two different methods.

### Method 1

“U.S. Food and Drug Administration – FDA”: M. Smoker, A. J. Krynitsky: “Interim Method for Determination of Melamine and Cyanuric Acid Residues In Foods using LC-MS/MS: Version 1.0”, link: http://www.cfsan.fda.gov/~frf/lib4422.html.

**Extraction and purification:** 5 g of sample is weighed, 20ml of mixture of acetonitrile/water is added (50/50,%(v/v)) and it is centrifuged at 5 000rpm for 20 minutes. Then the extract is purified using Waters Oasis MCX SPE cartridges according to the procedure described in the method. The eluate is evaporated and dissolved in 1ml of mixture acetonitrile/water (50/50,%(v/v)). The prepared sample is injected in the liquid chromatograph coupled with mass detector (LC/MS/MS method).

**Measurement conditions:** The ion source is electrospray ionisation in positive mode, ESI+, mobile phase: mixture of 10mM ammonium acetate in water and 10mM ammonium acetate in acetonitrile, flow rate of mobile phase 0.5ml/min, total analysis time 12 minutes, XBridge HILIC column, elution with optimized gradient.

**Concentration calculation:** Using the standard addition method.

### Method 2

From WATERS application note: J. Shia, C. Mallet, M. Young, J. Li, Y. Meng, C. Qi, “Rapid, specific analysis of melamine contamination in infant formula and liquid milk by UPLC/MS/MS”, link: http://www.sepscience.com/getattachment/fb78652b-eb85-43fd-8eb4-152cb9af2299/.aspx

**Extraction and purification:** 0.5 g of sample is extracted with 10 ml of 0.2 M perchlorate acid. The sample is sonified for 10 minutes in ultrasonic bath and then it is centrifuged at 5000 rpm for 10 minutes. The sample purification is performed using Waters Oasis MCX SPE cartridges, according to the procedure specified in the application note.

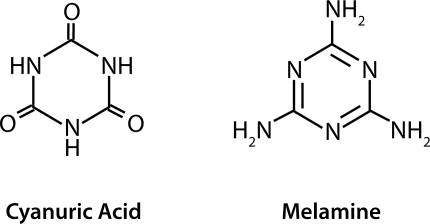



**Measurement conditions:** They are same as for the first method.

**Concentration calculation:** Using the standard addition method.

### Determination of cyanuric acid

The same preparation procedure (extraction with 0.2 M perchlorate acid, ultrasonic bath and centrifuge) was used and purification was performed using Waters Oasis MAX SPE cartridges. The purification procedure is described in the method “Interim Method for Determination of Melamine and Cyanuric Acid Residues In Foods using LC-MS/MS: Version 1.0”.

**Measurement conditions:** Electrospray ionisation in negative mode ESI- is used; chromatographic conditions are the same as for melamine determination.

**Concentration calculation:** Using the standard addition method.

## Remark on methods

For melamine determination the method of extraction to acetonitrile was initially used. This method was preliminary tested in the laboratory and its performance was very good, e.g. for the dried milk sample. However, as the subject of the analysis, the rice and the pea protein concentrates behaved in a very atypical fashion and extraction with acetonitrile was not efficient for this material type. It was not even possible to detect satisfactory melamine added as standard. Therefore we decided to try a stronger method – extraction with perchlorate acid. This extraction procedure was also used to determine cyanuric acid. Recoveries for both analytes were around 70%.

## Current incidence of melamine and cyanuric acid in food

Increased concern about melamine in food appeared with respect to its content in the milk that caused serious diseases, even deaths of infants in China. There were more than 52 thousand cases reported in China (some sources mention 94 thousand) with a diagnosis of kidney disease. Melamine was intentionally added to milk to adulterate a higher protein content in milk after dilution with water. Chinese authorities reported up to 294 thousand sick children in connection with milk contamination by melamine. In addition to infant food, melamine was found in many milk products. Incidence of contaminated food was reported in New Zealand, Taiwan, South Korea, Congo and elsewhere. In Europe, incidence of melamine was reported in Austria, Finland, Germany, Slovakia and in Hungary. In the Czech Republic, the official incidence of melamine in food by inspection bodies was not confirmed on any large scale. Exceptions were, for example, Emarko sticks containing excess melamine that were withdrawn from the market, and Chinese ‘White Rabbit’ sweets which practically were not distributed. Generally, melamine as a contaminant was already found in many foods throughout the world, and not only in dairy products, e.g. yoghurts, but also in various chocolates and cookies. In the field of legislation these problems are dealt with in the Official Journal of European Union by the Commission Decision of 14 October 2008, which imposes special conditions governing the import of products containing milk or milk products originating in or consigned from China, which stipulates that when the melamine level of 2.5 mg/kg is exceeded, products shall be destroyed immediately. This precaution is also valid for all products where the above concentration was found.

## Current incidence of melamine and cyanuric acid in feed

In China melamine was also added intentionally to vegetable protein concentrates to increase the content of nitrogen compounds (crude protein) in these products. The first problems appeared in 2007 in the USA in the form of the mass death of cats and dogs caused by renal failure. The cause was contaminated gluten added as protein concentrate to feed (Dobson, 2008). The diagnosis was melamine. Melamine contamination of feed caused the death of four thousand dogs and cats. In total, there were 1 950 cases of cats and about 2200 cases of dogs reported dead. Deaths of dogs were also reported from the Republic of South Africa. The cause was gluten (contaminated with melamine) used for feed production. It was the mass death of cats and dogs after feed intake where high concentrations of melamine were found that drew attention to the toxic effects of melamine. The cause was acute renal failure (Brown *et al*., 2007). Histopathologiocal, toxicological and clinical-pathological changes like uremia, anorexia, vomiting, lethargy, polyuria, azotemia and hypophosphatemia were described in these dogs and cats. In the distal tubules or collecting ducts of sick and dead animals specific polarizable crystals were present. In some animals chronic histological changes characterized by interstitial fibrosis and inflammation were found. In cats after intake of feed contaminated with melamine and cyanuric acid, clinical symptoms such as a refusal to eat, vomiting, polyuria, excessive thirst and lethargy were recorded. Histological research of kidney samples from these cats found intratubular crystalluria, tubular necrosis with degeneration and subcapsular perivascular inflammation with sysmptoms of perivascular fibroplasia or fibrosis and inflammation with intravascular fibrinous thrombosis. Toxicological analyses found the presence of melamine and cyanuric acid in the samples of cat feed, in vomitus, in urine and in the kidneys (Puschner *et al*., 2007). Clinical observations of acute renal failure in cats and dogs were connected with the intake of feed in which wheat gluten originating from China was used. Analyses of feed revealed the presence of simple triazine compounds, esp. melamine and cyanuric acid, together with lower concentrations of ammeline, ammelide, ureidomelamine and N-methylmelamine. Pathological findings in cats and dogs that died from acute renal failure indicated the presence of crystals in kidney tubules. Infrared microspectroscopy of individual crystals from the kidneys of cats or rats confirmed the presence of crystals composed of melamine and cyanuric acid. Crystals obtained from contaminated gluten produced comparable spectra. The withdrawal of feed for domestic animals in connection with acute renal failure in dogs and cats was originally focused on melamine as the main suspicious toxic substance. In the course of research the second toxic compound cyanuric acid was identified in addition to melamine (Cianciolo *et al*., 2008). From the point of view of safety, most reports state that products from livestock fed by contaminated feed pose only a slight risk to human beings. Nevertheless, recently melamine was even found in eggs originating from China, where contaminated feed is considered as its source. Examples of potentially hazardous raw materials in feed production are wheat aleurone, rice flour, corn flour, soya protein and other soya products, rice pollard, rice protein concentrate etc.

## The respective incidence of melamine and cyanuric acid in feed

**Figure 1:**
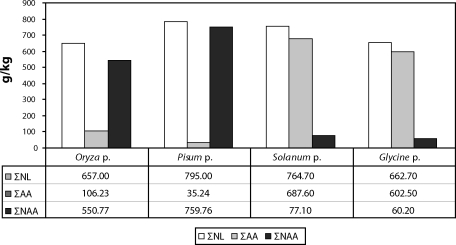
Content of nitrogen compounds (NL), aminoacids (AA) and non-aminoacid nitrogen compounds (NAA) in analysed protein concentrates.

In this work we present two incidences of feed contaminated with melamine and cyanuric acid. They were two protein concentrates originating from China: one rice (*Oryza* p.) and one pea (*Pisum* p.). A minimum protein content of 45% (pea) and 60% (rice) were declared. These protein concentrates were delivered to our laboratory to verify the declared content of nitrogen compounds (N x 6.25). The analyses conducted confirmed the high content of nitrogen compounds (NL) in the delivered samples of feed. Based on the results, it could be expected that the content of aminoacids would correspond to the content of nitrogen compounds. However, a comparison of results was surprising since the content of nitrogen compounds did not correspond to the expected content aminoacids as is usual for similar feeding components, e.g. for potato protein (*Solanum* p.) or soya protein (*Glycine* p.), as shown in Figure 1.

**Figure 2:**
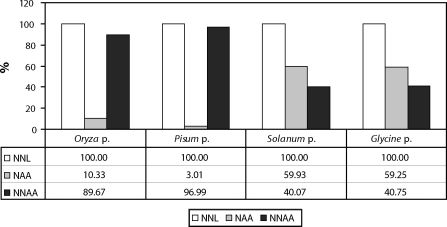
Percentage representation of aminoacid nitrogen (NAA) and of non-aminoacid nitrogen (NNAA) from total nitrogen (NNL) in analysed protein concentrates.

It follows from Figure 1 that in the suspicious protein concentrates (rice and pea concentrates), in spite of the high content of nitrogen compounds, there is a low content of total aminoacids and a very high content of other nitrogen compounds. On the contrary, common protein concentrates, like those of potato and soya, are characterized by their high content of total aminoacids and low content of other non-protein nitrogen compounds. Analysis of the rice and the pea protein concentrates revealed that the increased content of nitrogen compounds was caused by added melamine and cyanuric acid. In the rice protein the content of melamine was 3 200 mg/kg and 3 096 mg/kg for cyanuric acid; for the pea concentrate the melamine content was 3 420 mg/kg and 3 260 mg/kg for cyanuric acid. In reality, higher contents of these compounds can be expected since the recovery of the methods used is 70%. In the potato and the soya protein concentrates, melamine and cyanuric acid were not found. It can be concluded from the results that in the rice and the pea proteins the high contents of nitrogen in the determined nitrogen compounds are caused by non-protein nitrogen, as documented in Figure 2.

It follows from Figure 2 that in the rice protein there is only 10.33% and in the pea protein only 3.01% of nitrogen bound in aminoacids, whereas nitrogen in other nitrogen compounds represents 89.67% and 96.99%, respectively. In the potato and the soya concentrates almost 60% of nitrogen was bound in aminoacids, while only 40% of nitrogen was bound in other nitrogen compounds.

## Conclusions

It can be concluded from the results that protein concentrates are the main risk factor of food chain contamination with melamine and cyanuric acid, especially those of unknown origin or imported from countries outside the EU. Feed with a high content of nitrogen compounds and a low content of aminoacids can be regarded as particularly suspicious. Comparison of test results to determine nitrogen compounds and amines can be used as proof of adulteration of protein feeds. These feeds must be subjected to further analysis to determine the presence of melamine and cyanuric acid.
